# Stereo reconstruction from microscopic images for computer-assisted ophthalmic surgery

**DOI:** 10.1007/s11548-024-03177-0

**Published:** 2024-06-04

**Authors:** Rebekka Peter, Sofia Moreira, Eleonora Tagliabue, Matthias Hillenbrand, Rita G. Nunes, Franziska Mathis-Ullrich

**Affiliations:** 1 Carl Zeiss AG, Oberkochen, Germany; 2 Laboratory for Surgical Planning and Robotic Cognition (SPARC), Department of Artificial Intelligence in Biomedical Engineering, Friedrich-Alexander-Universität Erlangen-Nürnberg, Erlangen, Germany; 3 Institute for Systems and Robotics, Instituto Superior Técnico, Universidade de Lisboa, Lisbon, Portugal

**Keywords:** Stereo reconstruction, Computer-assisted ophthalmic surgery, Distortion correction, Depth estimation

## Abstract

****Purpose**:**

This work presents a novel platform for stereo reconstruction in anterior segment ophthalmic surgery to enable enhanced scene understanding, especially depth perception, for advanced computer-assisted eye surgery by effectively addressing the lack of texture and corneal distortions artifacts in the surgical scene.

****Methods**:**

The proposed platform for stereo reconstruction uses a two-step approach: generating a sparse 3D point cloud from microscopic images, deriving a dense 3D representation by fitting surfaces onto the point cloud, and considering geometrical priors of the eye anatomy. We incorporate a pre-processing step to rectify distortion artifacts induced by the cornea’s high refractive power, achieved by aligning a 3D phenotypical cornea geometry model to the images and computing a distortion map using ray tracing.

****Results**:**

The accuracy of 3D reconstruction is evaluated on stereo microscopic images of ex vivo porcine eyes, rigid phantom eyes, and synthetic photo-realistic images. The results demonstrate the potential of the proposed platform to enhance scene understanding via an accurate 3D representation of the eye and enable the estimation of instrument to layer distances in porcine eyes with a mean average error of 190 $\upmu {\hbox {m}}$, comparable to the scale of surgeons’ hand tremor.

****Conclusion**:**

This work marks a significant advancement in stereo reconstruction for ophthalmic surgery by addressing corneal distortions, a previously often overlooked aspect in such surgical scenarios. This could improve surgical outcomes by allowing for intra-operative computer assistance, e.g., in the form of virtual distance sensors.

**Supplementary Information:**

The online version contains supplementary material available at 10.1007/s11548-024-03177-0.

## Introduction

Cataract is the leading cause of blindness globally [1] and can be treated surgically. Cataract surgery requires extensive training and precision to access and remove the clouded lens. It is a delicate procedure that requires manipulating eye structures in the micrometer range. The small surgical field and transparent tissues make it difficult for surgeons to accurately judge depth and spatial relationships.

Modern digital ophthalmic microscopes providing digitized stereo image pairs open the door to stereo reconstruction. A stereo reconstructed 3D model of the patient eye and surgical instruments could enhance scene understanding and enable intra-operative computer assistance in the form of virtual distance sensors [2] to compensate for limited depth perception.

**Fig. 1:**
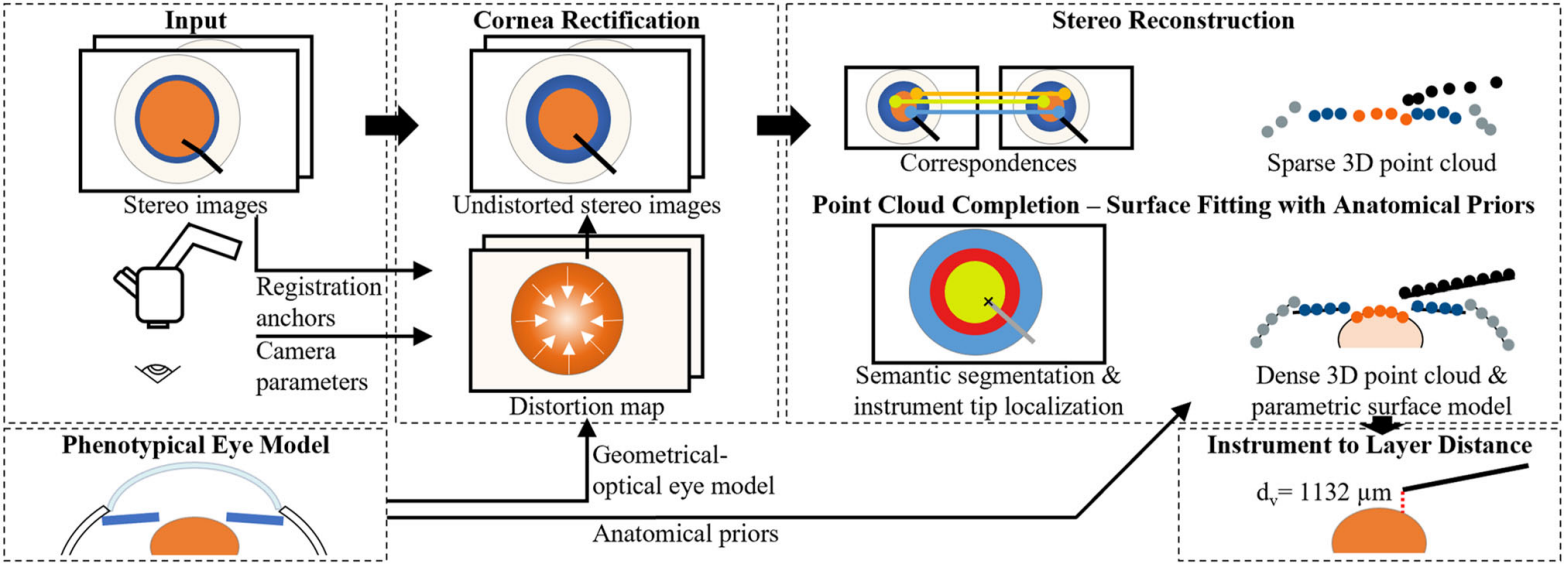
Our proposed platform for stereo reconstruction from microscopic images for computer-assisted ophthalmic surgery

Stereo reconstruction is a researched area in the medical field. Both supervised deep learning (DL) methods [3] and unsupervised approaches considering left–right consistency [4] have been developed to handle challenges in medical data, such as homogeneous areas and reflections. Little research exists in the field of stereo reconstruction for anterior eye segment surgery. Challenges include semi-transparent structures, homogeneous areas, strong light reflections, and distortion artifacts caused by the high refractive power of the cornea. In the field of ophthalmic surgery, the feasibility of reconstructing the retinal surface in the posterior eye segment from microscopic images has been demonstrated through methods such as semi-global block matching [5]. Probst et al. [6] apply semi-dense matching methods to generate correspondences and reduce noise and outliers by fitting a B-Spline surface on the reconstructed retinal points. However, none of these works take into account the distortion effects of the cornea and lens, and accordingly methods have only been validated in simplified open-sky scenarios (i.e., on eyes with removed cornea).

The high refractive power of the eye’s optical structures can cause misleading depth perception with stereo vision and violate the triangulation principle on which stereo reconstruction is based. Refraction effects on the cornea cause structures below, such as the lens and iris, to appear magnified, as shown in Online Resource 1.

Related work for stereo reconstruction with geometrically distorted images is found in the field of reconstruction of underwater scenes. In particular, refraction effects have been modeled as lens distortion [7] and as a focal length change [8]. These models are based on approximations and do not generalize to non-planar or multi-surface refraction like the cornea. Pável et al. [9] use neural networks in combination with ray tracing to render undistorted images, requiring distortion-free views of the training data. Unsupervised DL-based methods proposed for image undistortion tasks [10] require features that are absent in microscopic eye images, such as straight lines, limiting their applicability to eye surgery.

In this work, we present a novel platform for stereo reconstruction for anterior eye segment surgery. To address the lack of texture in the homogenous areas of the scene, we propose a two-step approach: (1) We generate a sparse 3D point cloud by identifying suitable features from the microscopic images. (2) We derive a dense 3D representation by fitting surfaces onto the point cloud while considering geometrical priors of the eye anatomy. To reduce the errors induced by cornea refraction, we include the rectification of the distortion artifacts as a pre-processing step in our platform. We utilize ray tracing, employing a phenotypical model of the eye geometry.

Our contributions include the development of a stereo reconstruction platform specialized for anterior ophthalmic surgery, which considers for the first time the distortion effects apparent in microscopic images of the eye. We evaluate the accuracy of 3D reconstruction and 3D instrument to layer distance sensing on microscopic images of ex vivo porcine eyes, rigid phantom eyes and synthesized photo-realistic images.

## Methods

Figure 1 illustrates the details of our platform. The input consists of rectified left and right camera images acquired from a calibrated surgical microscope, displaying the anterior segment of the eye from a top-down perspective during cataract surgery. Our approach addresses the situation in which a surgical instrument is inserted through the cornea and into the anterior eye chamber to manipulate the lens and lens capsule. An approximate alignment of the imaged eye to the optical axis of the microscope’s camera is required.

### Rectification of cornea distortion artifacts

To correct image distortions induced by the light refraction on the corneal surfaces, we create a geometrical-optical model of the scene, which includes the patient eye and the cameras of the surgical microscope. Using ray tracing, we compute a distortion map that represents the refraction effect on the corneal surfaces.

**Geometrical-optical cornea model: ** As patient-specific geometrical and optical characteristics cannot be directly obtained from available data, we approximate them using phenotypical eye models from literature for human [11] and porcine eyes [12]. The distance between imaged features and cornea affects the distortions and is not known either. Due to the depth ambiguity, it cannot be determined without first correcting for the cornea distortions. To overcome this, we estimate the distortion map for image points in the pupil plane as an approximation of the average depth of interest.

**Registration of the eye model to the images: ** Assuming approximate alignment of the eye with the camera’s optical axis and rotational symmetry of the 3D phenotypical eye model, the Degree of Freedom (DoF) for the registration of the eye model with the 2D images is reduced to three-dimensional translation. The central point of the anterior lens surface is used as a 3D anchor point. As the camera rays intersect the cornea almost orthogonally, we hypothesize that the corneal distortion artifacts are minimal at the center, following Snell’s law. Consequently, we can estimate the center point’s 3D position using the stereo reconstruction principle. The center point of the lens in the left image is manually annotated. To determine its disparity and from this, the 3D coordinates, we use single-pixel block matching (BM).

**Computation of the distortion map using ray tracing: ** The cornea distortion map is computed using ray tracing. A ray tracing renderer casts the camera rays on the scene composed of the virtual image plane, two corneal surfaces, and the approximate pupil plane. After obtaining the intersection between the refracted rays and the pupil plane, the re-projection of these points in the virtual image plane $(u', v')$ is computed using a pinhole camera model without any refractive surfaces along the ray path. Subtracting the original pixel coordinates (*u*, *v*) from the re-projected pixel coordinates $(u', v')$ gives the distortion map. Using the distortion map and linear interpolation, the image is remapped to rectify the corneal distortion artifacts. The full process is executed for the left and right camera individually.

### Stereo reconstruction

Stereo reconstruction creates depth perception from two images captured from different perspectives. This process necessitates a calibrated camera system and rectified images. By matching corresponding pixels in the vertically aligned left and right images, the disparity $d = u_{left} - u_{right}$ is determined. The disparity directly correlates with the distance *z* from the camera to the point on the scene, with $z(d) = \frac{f \times b}{d}$, where *f* is the focal length of the rectified camera system and *b* is the baseline, i.e., the distance between the left and right camera center.

**Sparse 3D point cloud: ** We establish sparse correspondences between the left and right images using traditional feature detection and matching algorithms, namely scale-invariant feature transform (SIFT) [13] and Fast Library for Approximate Nearest Neighbors (FLANN) [14]. To enhance feature point detection in areas such as the iris and lens, we fine-tune the SIFT algorithm parameters and, in particular, significantly reduce the contrast threshold. False-positives are eliminated using a ratio test with the FLANN matcher [13]. Additionally, all matched features are removed that are not along the same horizontal line, with a tolerance of $\pm 5$ pixels, as they violate the epipolar constraint of rectified images.

**Point cloud completion - surface fitting with anatomical priors: ** The obtained 3D point cloud is sparse due to the lack of texture in the scene. We propose a point cloud completion strategy that leverages knowledge about the phenotypical eye geometry. For that, the different anatomical structures of the eye are segmented and for each structure, a surface is fitted onto the corresponding 3D points, see Online Resource 2.

We semantically segment the 3D point cloud manually into four sets, namely the sclera, iris, lens, and the surgical instrument. We reduce the problem of segmenting the point cloud to the simpler problem of segmenting the left camera image.

Four individual parametric surfaces are then fit onto the four segmented sets of 3D points while applying Random Sample Consensus (RANSAC) for outlier removal. Motivated by [11], a spherical surface is used for the sclera, while conical surfaces are used for the lens and iris. Conical surfaces are chosen to represent the iris, based on empirical observations of 3D sparse point clouds and Optical Coherence Tomography (OCT) scans. The surgical instrument is represented by a line. Once the 3D shape of the eye segments is obtained using surface fitting, the image pixels are projected onto the parametric surfaces to visualize a dense and colored point cloud.

**Fig. 2:**
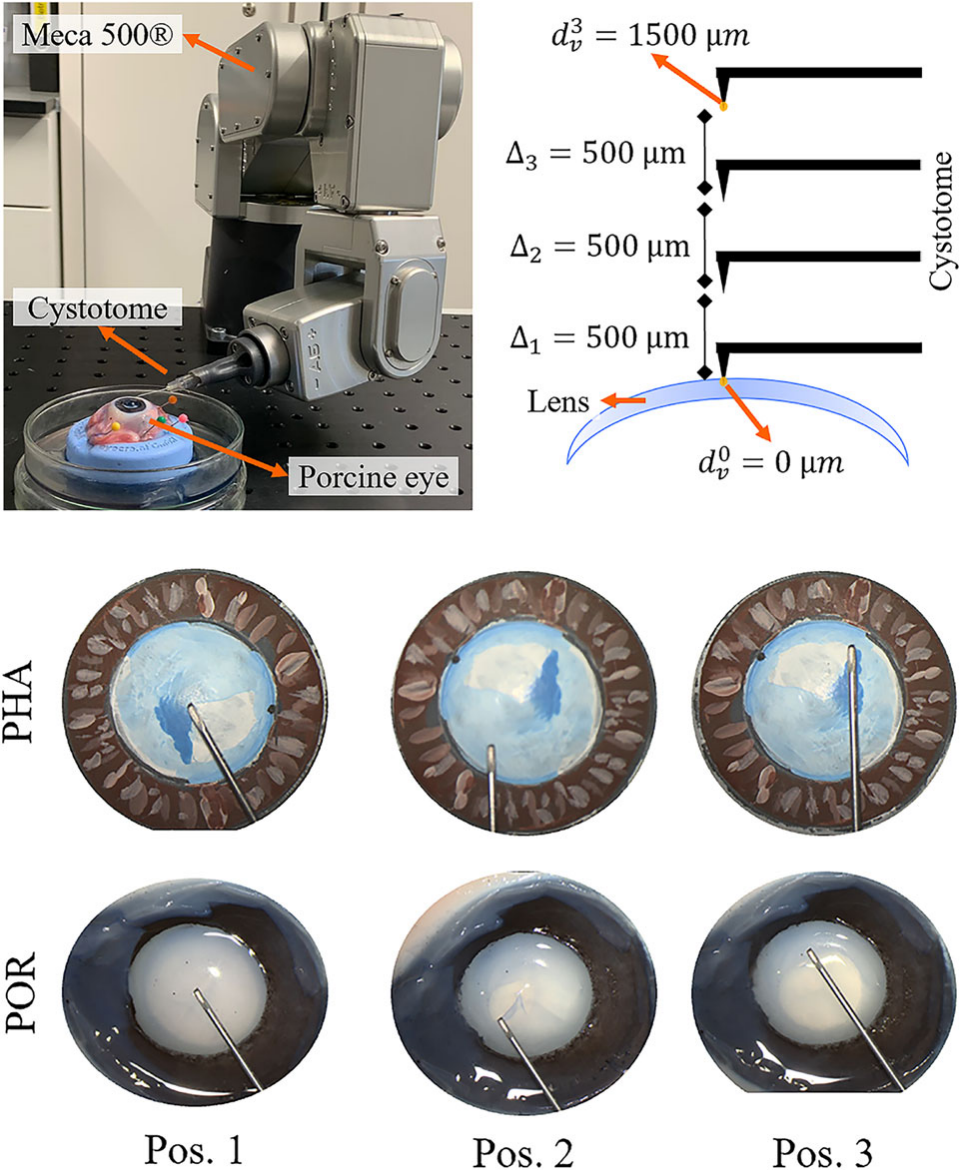
Experimental setup for vertical distance $d_v$ estimation between instrument and lens

**Instrument to layer distance: ** To estimate the vertical distance between the tip of a surgical instrument and the lens surface, we manually annotate the instrument tip in the left image. Using single-pixel BM, we determine the corresponding point in the right camera image. Based on the disparity and camera intrinsics, we calculate the 3D instrument tip position $(x_i, y_i, z_i)$. We then compute the vertical distance $d_v$ to the parametric surface of the lens $f_{lens}(x,y)$ as $d_v = z_i - f_{lens}(x_i, y_i)$.

## Experimental evaluation

### Experiment outline

To evaluate the performance of the proposed platform, two sets of experiments are conducted: The first focuses on stereo reconstruction, with the assumption that cornea distortion artifacts are corrected. We perform two sub-experiments: Experiment 1a compares the geometry derived from our approach with reference geometry of the test object. Experiment 1b evaluates the accuracy of distance estimation between a surgical instrument tip and the anterior lens surface via stereo reconstruction.

Figure 2 shows the experimental setup for experiment 1b. Using a Meca 500 robotic manipulator, a cystotome, a commonly used instrument in cataract surgery, is methodically elevated by 500 $\upmu {\hbox {m}}$ until it reaches a distance of 1500 $\upmu {\hbox {m}}$ from the starting point. A stereo pair is captured after each increment. The starting position, where the cystotome tip contacts the lens surface, sets a reference distance of 0 $\upmu {\hbox {m}}$.

In the second experiment, we evaluate the influence of the cornea distortion artifacts on the reconstruction and the effectiveness of our correction approach (experiment 2a). For experiment 2b, we consider the scenario that the reference geometry of the cornea is not precisely known.

### Data acquisition

We collect data from ex vivo porcine eyes (POR) and a phantom eye (PHA) using a calibrated ZEISS KINEVO 900 surgical microscope (Carl Zeiss Meditec AG, Germany).

**Ex-vivo porcine eyes (POR): ** Stereo images of ex vivo porcine eyes are captured during continuous curvilinear capsulorhexis (CCC), a step of cataract surgery. In this step, the capsule bag, a thin membrane surrounding the lens, is opened by tearing it with a surgical needle (cystotome). The images from ex vivo porcine eyes exhibit lower visual quality compared to images from human eyes during cataract surgery due to the absence of a red reflex from the retina. During cataract surgery, a specialized ophthalmic microscope is employed which exhibits more structure. To enhance contrast during capsulorhexis on the porcine eyes, we use Trypan Blue to stain the lens capsule [15]. Variations of test data are created by inducing cataracts in ex vivo porcine eyes through microwaving until the lens becomes cloudy (approx. 10s at 700W), as detailed in [16]. The purpose of this is to mimic a more realistic visual appearance. To assess the stereo reconstruction pipeline independently of the cornea’s influence, we also capture images from three eyes with the cornea removed.

**3D-printed phantom eye (PHA):** We reference the average human eye geometry [11] to design a geometrical model of the eye. We utilize a high-precision Form 3+ resin printer (Formlabs GmbH, Germany) to 3D print this model, excluding the cornea. To emulate the appearance of an eye during cataract surgery, the model is textured with a thin layer of acrylic paint.

**Synthetic data with photo-realistic rendering (SYN):** We employ the same geometrical model based on [11] as used for the 3D-printed phantom eye to create a digital eye model. With ray tracing, we generate stereo pairs, taking into account the intrinsic and extrinsic camera parameters of the ZEISS KINEVO 900 surgical microscope’s full resolution of $1920\times 1080$ pixels. The rendering process incorporates the refraction of light rays at the cornea according to Snell’s law [17]. We consider refractive indexes from literature [18]. The surfaces of the 3D models are textured in Blender using texture from various microscopic data of human eye structures.

**Fig. 3:**
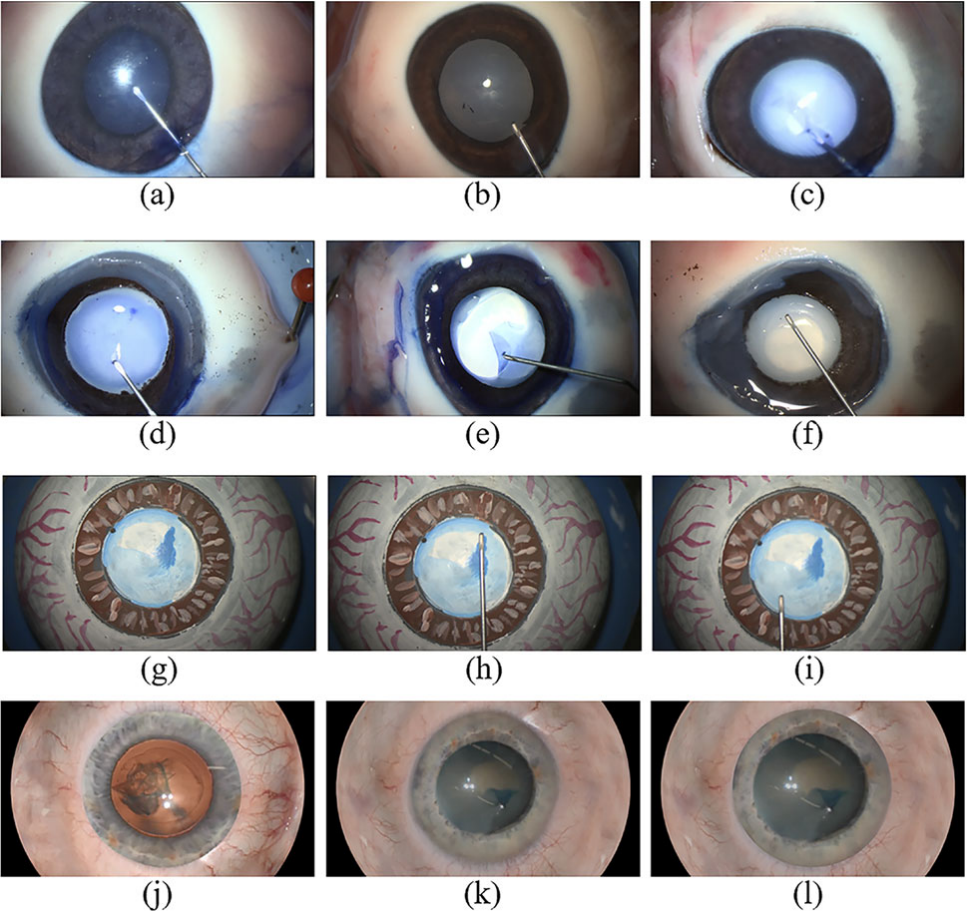
Overview of acquired data. **a**–**f** POR, **g**–**i** PHA, **j**–**l** SYN

Figure 3 presents examples of the acquired data. Images of porcine eyes that include a cornea (a–c) are either blurred due to the use of microwaving to induce a cataract, or lack lens surface features because of the absence of the red reflex in the neurosurgical microscope. The images acquired from porcine eyes without cornea (d–f) provide a more robust and realistic dataset for testing the stereo reconstruction algorithms. While the visual and geometrical features of the phantom eye (g–i) are simplified compared to human eyes, a precise geometrical reference is available. Figure 3 further shows the synthesized images without cornea (j–k). Image (l) corresponds to image (k), including the corneal distortion. The red reflex from the retina is visible in (j).

### Evaluation metrics

To evaluate the accuracy of our stereo reconstruction method in experiment 1a and 2, we compare the reconstructed 3D coordinates of every image pixel with their reference 3D coordinates. The primary metric employs the Euclidean distance between the reconstructed and reference positions, analyzed in form of the mean average error (MAE) across all pixels. For SYN data, the reference 3D position for every image pixel is available. For PHA data, the reference geometry of the eye is available, but the absolute position is unknown. Consequently, the reconstructed point cloud is aligned to the reference volume using RANSAC first. For POR data, the lack of reference geometry allows only a qualitative evaluation of the reconstructed geometry. This evaluation assesses consistency and whether different stereo images from the same porcine eye generated identical 3D models.

The instrument tip to lens distance estimation (experiment 1b) is evaluated using the vertical distance $d_0$ predicted for each sequence’s first stereo pair as reference. Relative distances $\Delta _1,\Delta _2,\Delta _3$ between consecutive stereo pair instrument tips are evaluated, with the known reference value assumed to be 500 $\upmu {\hbox {m}}$. The absolute error (AE) for each distance step $\Delta _i$ with $i=1,2,3$ is computed.

### Baseline algorithm: dense disparity map

We compare our stereo reconstruction approach with Semi-Global Matching (SGM) [19], a classical dense feature matching algorithm. We fine-tuned the parameters of SGM for improved performance on our data: Block size of 16 pixels, disparity range of [0, 256] pixels, speckle window size of 200, speckle range of 4. The disparity maps are refined using the weighted least squares (WLS) filter [20].

## Results

### Stereo reconstruction

Table 1 presents the MAE and standard deviation (SD) of the stereo reconstruction. For experiment 1b (open-sky scenario), our method significantly outperforms SGM in both PHA and SYN data types. Our method keeps both MAE and SD under 70 $\upmu {\hbox {m}}$ for SYN data. In particular, the baseline has a high SD of 985 $\upmu {\hbox {m}}$ and 164 $\upmu {\hbox {m}}$ for PHA and SYN eyes, respectively. For PHA data, the MAE of 118 $\upmu {\hbox {m}}$ is higher than for SYN data, assumably due to unaccounted imaging and calibration errors of the stereo microscope.

**Table 1:** Results of stereo reconstruction without and with cornea. All values are in $\upmu {\hbox {m}}$

Experiment	Data	Our approach		Baseline: SGM$^\textrm{a}$	
		MAE	SD	MAE	SD
1b: Open-sky scenario	PHA (w/o cornea)	118	64	611	985
	SYN (w/o cornea)	51	67	66	164
No rect. of corneal distort.	SYN (w/ cornea)	305	339	478	180
2a: Rect. of corneal distort.	SYN (w/ cornea)	73	54	205	1171

$^\textrm{a}$All points with an Euclidean distance $>2000\,\upmu {\hbox {m}}$ are not considered

**Fig. 4:**
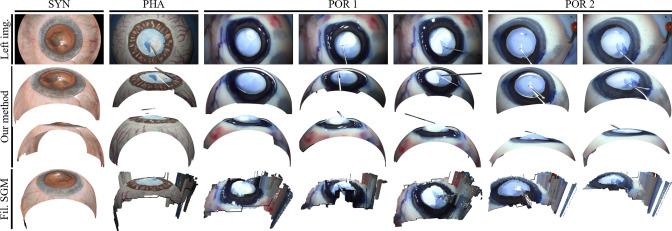
Stereo reconstruction results for SYN, PHA and POR

Figure 4 displays reconstruction results for selected examples within the three data types, including two different porcine eyes without cornea during CCC execution (POR 1 and POR 2). The reconstructed shape of POR 1 appears consistent across the three frames shown. However, the reconstruction of POR 2 is not visually plausible, with the lens appearing too flat in the second frame. The qualitative results confirm that our two-stage sparse-to-dense reconstruction approach clearly outperforms the baseline method of dense stereo reconstruction (filtered SGM). In Online Resource 3 and 4, further qualitative results are presented.

**Table 2:** Results of instrument to lens distance sensing experiment (1b) on a PHA and POR eye without cornea. All values are in $\upmu {\hbox {m}}$

		$\Delta _1$	AE(${\Delta _1}$)	$\Delta _{2}$	AE(${\Delta _2}$)	$\Delta _3$	AE(${\Delta _3}$)	MAE	MAE(Pos. 1-3)
	Pos. 1	674	174	463	37	467	33	80	
PHA	Pos. 2	680	180	702	202	796	296	226	145
	Pos. 3	528	28	679	179	324	176	128	
	Pos. 1	343	157	853	353	206	294	268	
POR	Pos. 2	257	243	669	169	732	232	215	194
	Pos. 3	471	29	335	165	398	102	99	

Table 2 displays the AE for the instrument tip to lens vertical distance estimation experiments. The experiment is repeated for a PHA and POR eye and three initial lateral instrument positions. On average, the achieved accuracy is 145 $\upmu {\hbox {m}}$ for the PHA sequences and 194 $\upmu {\hbox {m}}$ for the POR sequences, indicating less accurate stereo reconstruction for the porcine eye lens. The lower accuracy for POR data might result from a more complex geometry of porcine eyes that lead to a higher deviation from the fitted eye model.

### Rectification of cornea distortion artifacts

**Fig. 5:**
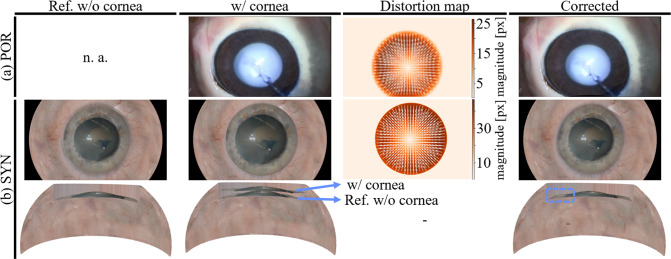
Rectification of corneal distortion. **a** Estimated distortion map for POR eye with cornea. **b** Distortion map for SYN data. The third row shows the reconstructed 3D models. Minor deviations are observed in the overlap with the reference geometry (blue rectangle)

Figure 5 shows the distortion maps for a POR and SYN image based on the estimated geometrical-optical model in the ray tracing scene. Distortion vectors point toward the center of the eye to contradict the magnification effect induced by the cornea. The magnitude of the distortion vectors is almost zero at the lens center but grows rapidly toward the periphery, with a maximum magnitude of 47 pixels for SYN. When rectifying the cornea distortion artifacts utilizing the distortion maps, the corrected images appear plausible (smaller pupil) and consistent. For the SYN data, the result visually matches the reference image from an eye without cornea. Figure 5b furthermore shows the reconstructed dense point clouds, with a cross-section view overlapped with the reference eye model. The reconstructed lens and iris are visibly shifted upwards for the distorted images. Applying the correction to the images overall recovers the reference position of the lens and iris with only minor deviations from the reference.

**Table 3:** Influence of a change in the geometrical and optical parameters of the ray tracing scene

10% increase of	$R_{ac}$	$k_{ac}$	$R_{pc}$	$k_{pc}$	$d_c$	$d_{ach}$	$n_{c}$	Ref	No rect. of corneal distort.
MAE	73	77	78	82	80	64	90	60	305
SD	110	115	113	116	115	108	117	53	339

MAE and SD of the stereo reconstruction of SYN data when the radius and conical constants of the anterior and posterior corneal surfaces $R_{ac}$, $k_{ac}$, $R_{pc}$, and $k_{pc}$, the corneal thickness $d_c$, the anterior chamber depth $d_{ach}$ and the refractive index of the cornea $n_c$ are varied by 10% individually. All values are in $\upmu {\hbox {m}}$

Table 1 displays the MAE and SD for stereo reconstructions for the scenarios of experiment 2a. The distortion correction strategy reduced MAE by >72% compared to reconstructing the stereo pair with cornea distortion artifacts.

Table 3 presents the reconstructed point clouds’ MAE and SD when varying the cornea model’s geometrical and optical parameters used for computing the distortion maps by 10% to mimic the variance between the phenotypical model and patient-specific cornea characteristics (experiment 2b). The MAE of the reconstruction does not increase by more than 30 $\upmu {\hbox {m}}$ compared to using the reference parameters. Even when inaccurate cornea geometries are assumed, the resulting MAE is significantly better than the MAE of 305 $\upmu {\hbox {m}}$ when not correcting for cornea distortion.

## Discussion

In this work, we create and employ a dataset of stereo microscopic images with known camera calibration of ex vivo porcine eyes, a phantom eye model and photorealistic synthetic data. Each data type presents its own set of limitations. Thus, different data are used for different experiments. The conclusions, when combined, provide a comprehensive assessment of the expected performance of our proposed platform for human eyes. Our proposed method for stereo reconstruction yields accurate reconstruction with a MAE below 120 $\upmu {\hbox {m}}$ of the eye anatomy and surgical instruments. Our approach significantly outperforms baseline experiments using dense stereo reconstruction, demonstrating the effectiveness of our method. Accuracy during ophthalmic surgery is generally limited by the surgeon’s hand tremor in the range of 100–140 $\upmu {\hbox {m}}$ [21]. Although the required accuracy strongly depends on the specific surgical step, concrete setup, and implementation, our proposed method shows errors in the same order of magnitude. The theoretical maximum depth resolution of the surgical microscope used for data acquisition is approximately 190 $\upmu {\hbox {m}}$. While this suggests potential for accuracy improvement, the results are reasonable when considering error propagation throughout the pipeline, particularly given the high system magnification and scene challenges. We show that without correction, the cornea distortion cause the MAE of the stereo reconstruction to be of an insufficient magnitude of 305 $\upmu {\hbox {m}}$ with a high standard deviation of 339 $\upmu {\hbox {m}}$. By utilizing our approach based on raytracing and registration, we are able to mitigate the distortion.

A major limitation of our approach lies in the use of simplified eye geometry models in synthetic and phantom eye data. However, results from experiment 2b, assessing the influence of changed or unknown corneal shape, suggest that our approach may accommodate variations between the average phenotypical eye model used and patient-specific eye geometry. Additionally, porcine eyes, which closely resemble more complex human eye geometry, yielded qualitatively accurate stereo reconstruction results.

Future work could address this situation by incorporating pre-operative data, higher-order parametrization of the surfaces, eye movement tracking for improved rectification and registration, or DL approaches. While our current setup requires user input in the form of manual annotation of the lens center and semantic segmentation, we anticipate that future iterations could automate these steps using DL-based computer vision pipelines. Pissas et al. [22] propose solutions for the required tasks for human eyes. They were not employed due to their inability to generalize to our test data, given the domain shift between porcine, phantom and human eyes. The current implementation is not optimized for real-time usage, which is essential during surgical procedures. In particular, the computationally expensive RANSAC algorithm used for outlier removal during surface fitting is one bottleneck. To optimize computational time, we plan to explore approaches such as downsampling image resolution, replacing the RANSAC algorithm, or utilizing a GPU.

## Conclusion

In this work, we propose a platform for accurate stereo reconstruction during anterior eye segment surgery using sparse feature matching and surface fitting. In previous works on stereo reconstruction for ophthalmic applications, corneal distortion was not considered and was left for future exploration. Our work takes a significant step in this direction by considering the distortion artifacts caused by the eye’s refracting surfaces and their impact on stereo reconstruction. Our contributions enable further research toward computer assistance and automation for ophthalmic surgery.

## Supplementary Information

Below is the link to the electronic supplementary material.

Supplementary file 1 (pdf 6542 KB)
