# Correction to: Stereo reconstruction from microscopic images for computer-assisted ophthalmic surgery

**DOI:** 10.1007/s11548-024-03270-4

**Published:** 2024-11-26

**Authors:** Rebekka Peter, Sofia Moreira, Eleonora Tagliabue, Matthias Hillenbrand, Rita G. Nunes, Franziska Mathis-Ullrich

**Affiliations:** 1 Carl Zeiss AG, Oberkochen, Germany; 2 Laboratory for Surgical Planning and Robotic Cognition (SPARC), Department of Artificial Intelligence in Biomedical Engineering, Friedrich-Alexander-Universität Erlangen-Nürnberg, Erlangen, Germany; 3 Institute for Systems and Robotics, Instituto Superior Técnico, Universidade de Lisboa, Lisbon, Portugal

**Correction to: International Journal of Computer Assisted Radiology and Surgery** 10.1007/s11548-024-03177-0

In the original version of this article, figures 2 and 3 are displayed 4 times bigger than intended. The figures, including text in the figures, are unnecessarily large and disturb the overall layout. The Figures 2 and 3 should have appeared as shown below.

**Fig. 2:**
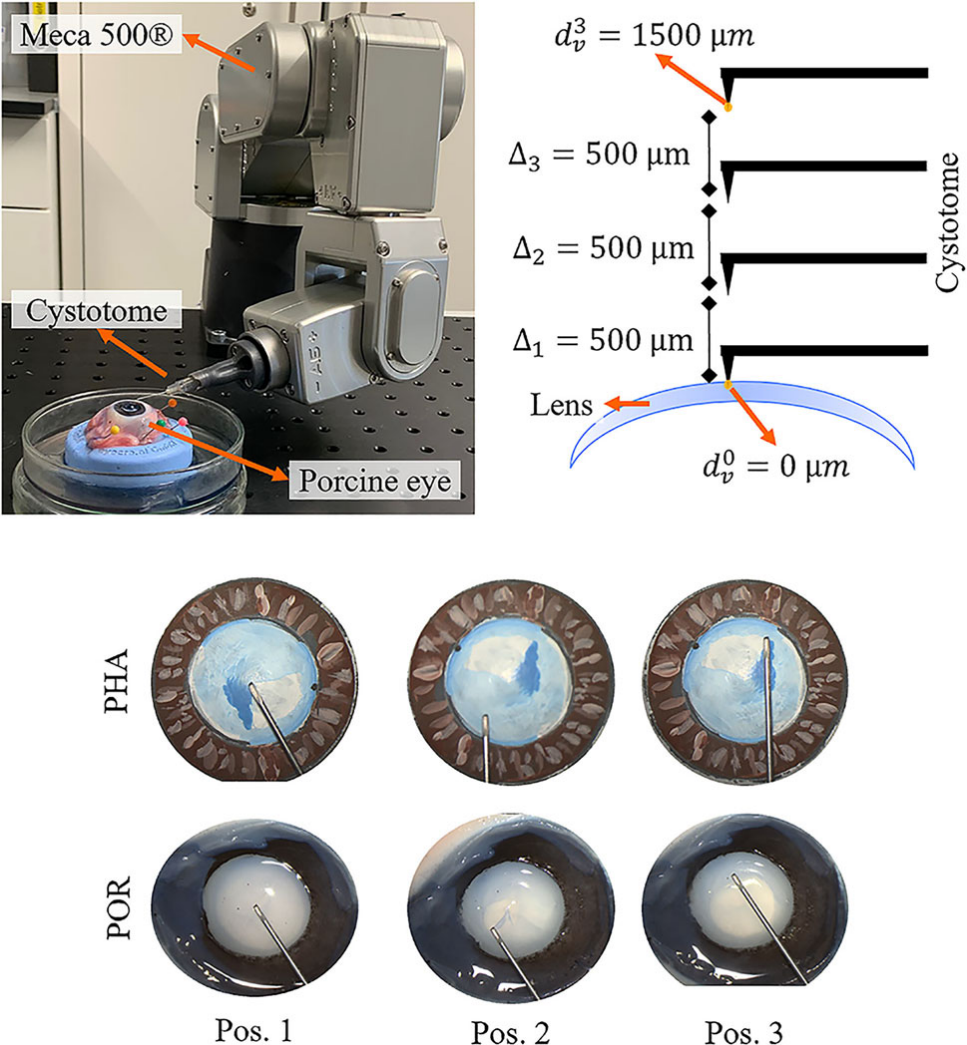
Experimental setup for vertical distance $d_v$ estimation between instrument and lens

**Fig. 3:**
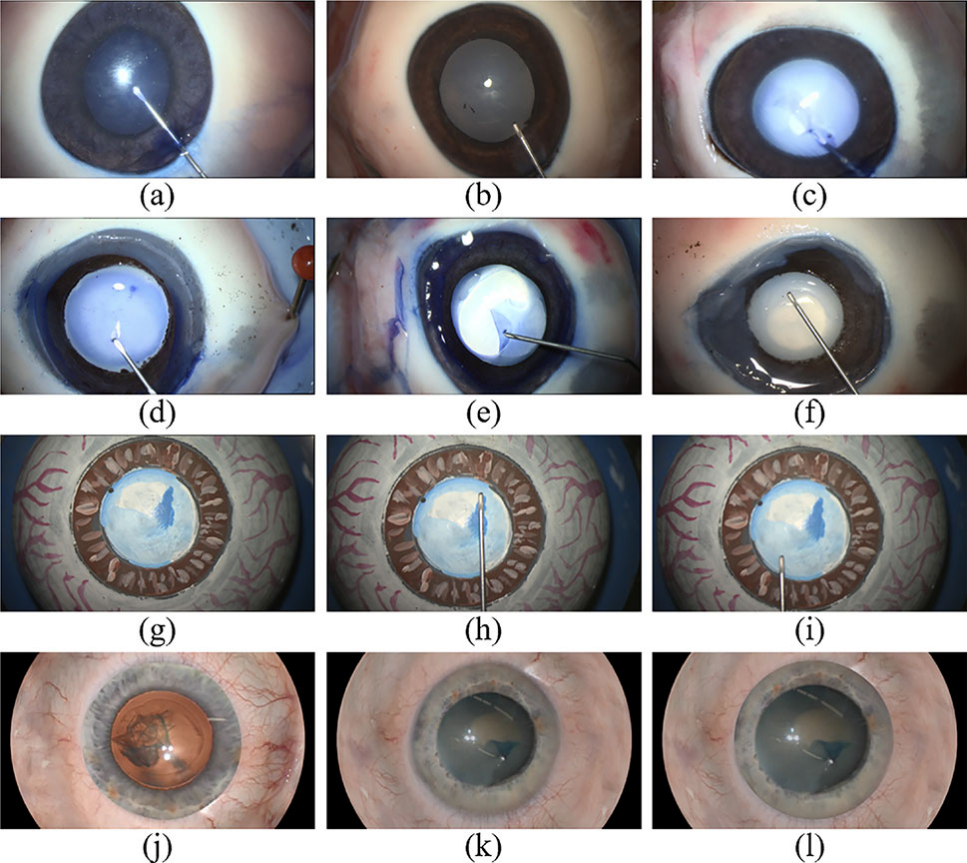
Overview of acquired data. **a**–**f** POR, **g**–**i** PHA, **j**–**l** SYN

The layout of the two tables is overall highly inconsistent. While for Table 1 the numbers are right-bound, for Table 2 the numbers are left-bound. For table 3 it is mixed. The alignment of the columns w. r. t. to the headlines is not clear and seems inconsistent as well. The added line between row 4/5 is not centered (closer to row 5 than to row 4). The column width is not equally distributed which leads to a unclear and unorganized overall appearance. The Tables 1, 2 and 3 should have appeared as shown below.

**Table 1:** Results of stereo reconstruction without and with cornea. All values are in $\upmu {\hbox {m}}$

Experiment	Data	Our approach		Baseline: SGM$^\textrm{a}$	
		MAE	SD	MAE	SD
1b: Open-sky scenario	PHA (w/o cornea)	118	64	611	985
	SYN (w/o cornea)	51	67	66	164
No rect. of corneal distort.	SYN (w/ cornea)	305	339	478	180
2a: Rect. of corneal distort.	SYN (w/ cornea)	73	54	205	1171

$^\textrm{a}$All points with an Euclidean distance $>2000\,\upmu {\hbox {m}}$ are not considered

**Table 2:** Results of instrument to lens distance sensing experiment (1b) on a PHA and POR eye without cornea. All values are in $\upmu {\hbox {m}}$

		$\Delta _1$	AE(${\Delta _1}$)	$\Delta _{2}$	AE(${\Delta _2}$)	$\Delta _3$	AE(${\Delta _3}$)	MAE	MAE(Pos. 1-3)
	Pos. 1	674	174	463	37	467	33	80	
PHA	Pos. 2	680	180	702	202	796	296	226	145
	Pos. 3	528	28	679	179	324	176	128	
	Pos. 1	343	157	853	353	206	294	268	
POR	Pos. 2	257	243	669	169	732	232	215	194
	Pos. 3	471	29	335	165	398	102	99

**Table 3:** Influence of a change in the geometrical and optical parameters of the ray tracing scene

10% increase of	$R_{ac}$	$k_{ac}$	$R_{pc}$	$k_{pc}$	$d_c$	$d_{ach}$	$n_{c}$	Ref	No rect. of corneal distort.
MAE	73	77	78	82	80	64	90	60	305
SD	110	115	113	116	115	108	117	53	339

MAE and SD of the stereo reconstruction of SYN data when the radius and conical constants of the anterior and posterior corneal surfaces $R_{ac}$, $k_{ac}$, $R_{pc}$, and $k_{pc}$, the corneal thickness $d_c$, the anterior chamber depth $d_{ach}$ and the refractive index of the cornea $n_c$ are varied by 10% individually. All values are in $\upmu {\hbox {m}}$

The original article has been corrected.

