# Supplementary material for: Stereo reconstruction from microscopic images for computer-assisted ophthalmic surgery
Source: Int J Comput Assist Radiol Surg. 2024 Jun 4;20(3):605–12. doi: 10.1007/s11548-024-03177-0 (PMC11929700; doi:10.1007/s11548-024-03177-0)
Supplement: Supplementary file 1 — (pdf 6542 KB) [file 11548_2024_3177_MOESM1_ESM.pdf]

Stereo Reconstruction from Microscopic Images for Computer-Assisted Ophthalmic Surgery

Rebekka Peter[1,2*†], Sofia Moreira[1,3†], Eleonora Tagliabue[1], Matthias Hillenbrand[1], Rita G. Nunes[3], Franziska Mathis-Ullrich[2]

[1*]Carl Zeiss AG, Oberkochen, Germany.
[2]Laboratory for Surgical Planning and Robotic Cognition (SPARC), Dep. Artificial Intelligence in Biomedical Engineering, Friedrich-Alexander-Universität Erlangen-Nürnberg, Erlangen, Germany.
[3]Institute for Systems and Robotics, Instituto Superior Técnico, Universidade de Lisboa, Lisbon, Portugal.

*Corresponding author. E-mail: rebekka.peter@zeiss.com;
†These authors contributed equally to this work.

Supplementary Information

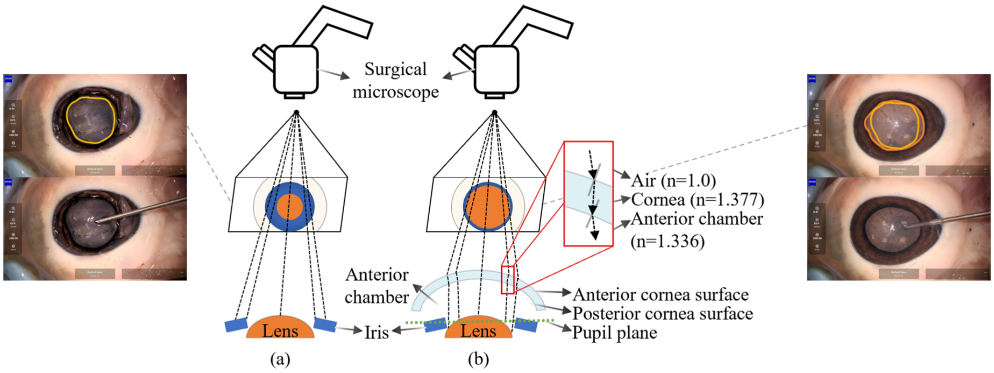

Online Resource 1: Impact of corneal refractive power on microscopic images of the eye. Due to the refraction on the corneal surfaces, the pupil appears magnified. The depth of imaged points influences the distortion in the image domain. We define the pupil plane as average depth of interest. In the microscopic images of porcine eyes, the edge of the pupil is highlighted in yellow (removed cornea) and orange (with cornea) to emphasize the magnification. An apparent bend in the surgical instrument is visible at the corneal incision.

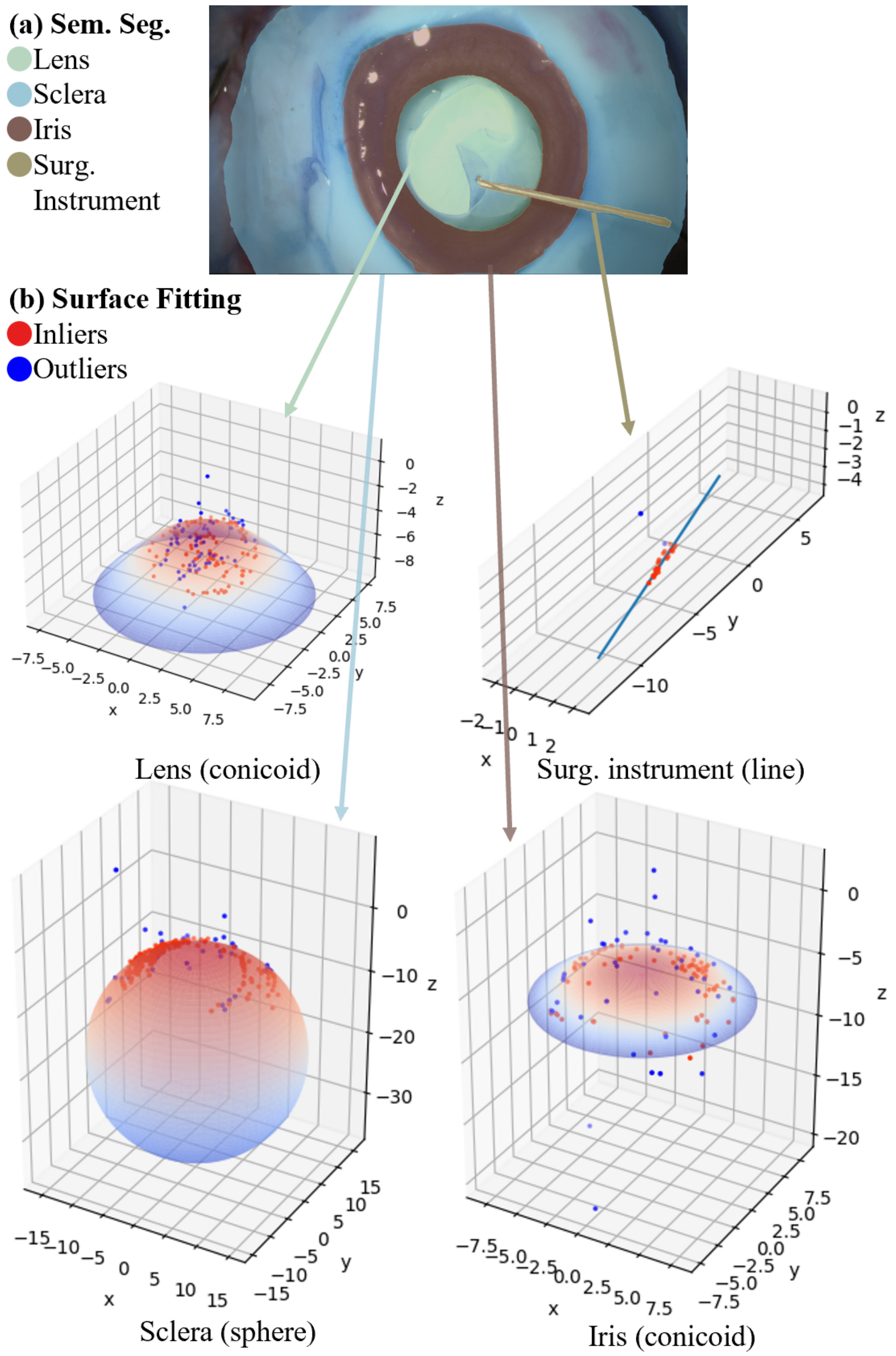

Online Resource 2: Overlay of a segmentation mask for a porcine eye microscopic image and surface fitting results of the four segments. The color of the surface encodes the height (z-direction).

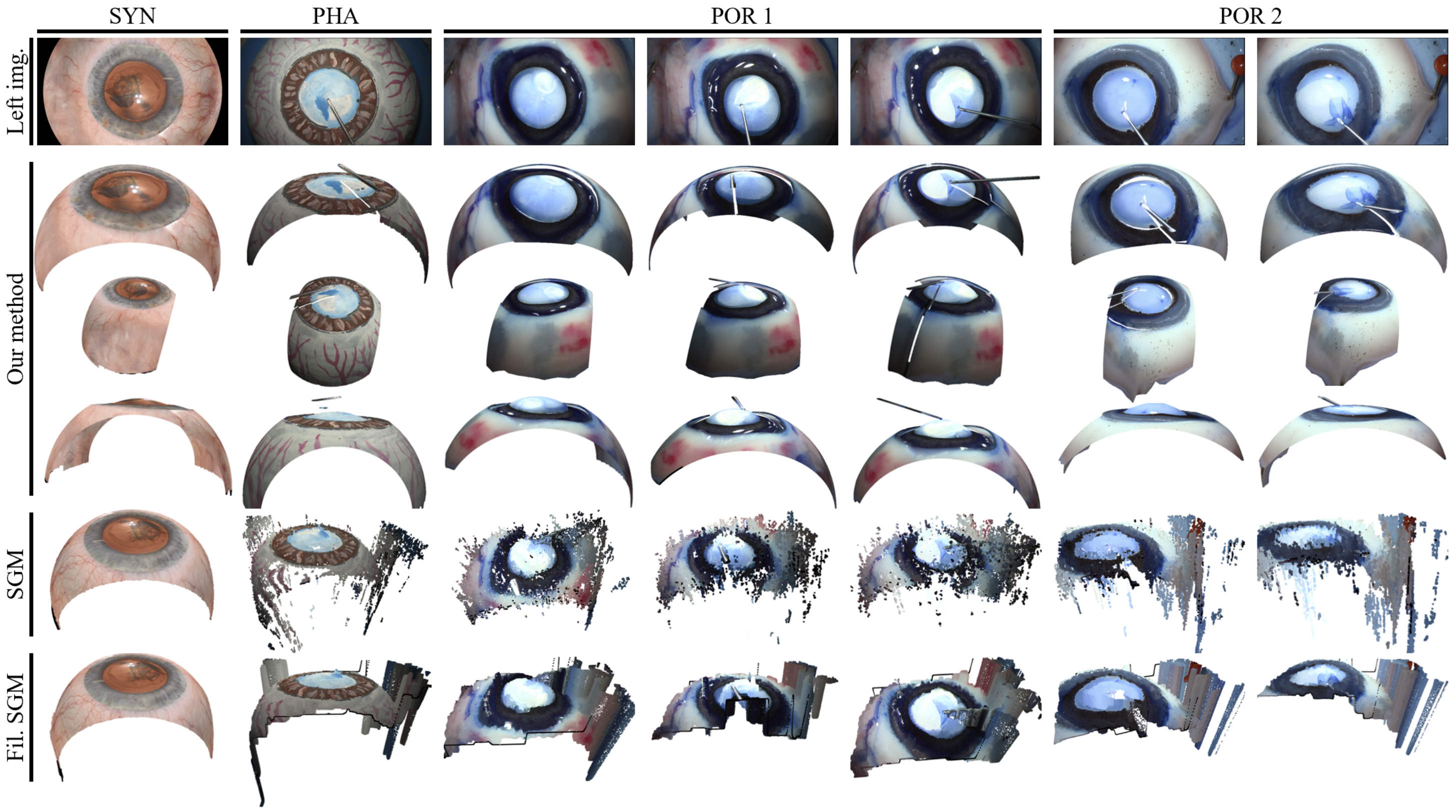

Online Resource 3: Stereo reconstruction results for SYN, PHA and POR. Row 1: Left image of the stereo pairs. Row 2-4: 3D model derived with our two-stage reconstruction approach from three perspectives. Row 5-6: 3D model derived with the baseline dense stereo reconstruction approaches Semi-Global Matching (SGM) and filtered SGM. Our approach yields plausible and consistent results for the SYN, PHA, and POR 1 data. POR 2 does not exhibit visually plausible results, with the lens appearing too flat in the second frame (row 4, last column). The SGM and filtered SGM 3D models are noisy and inconsistent for all data despite SYN.

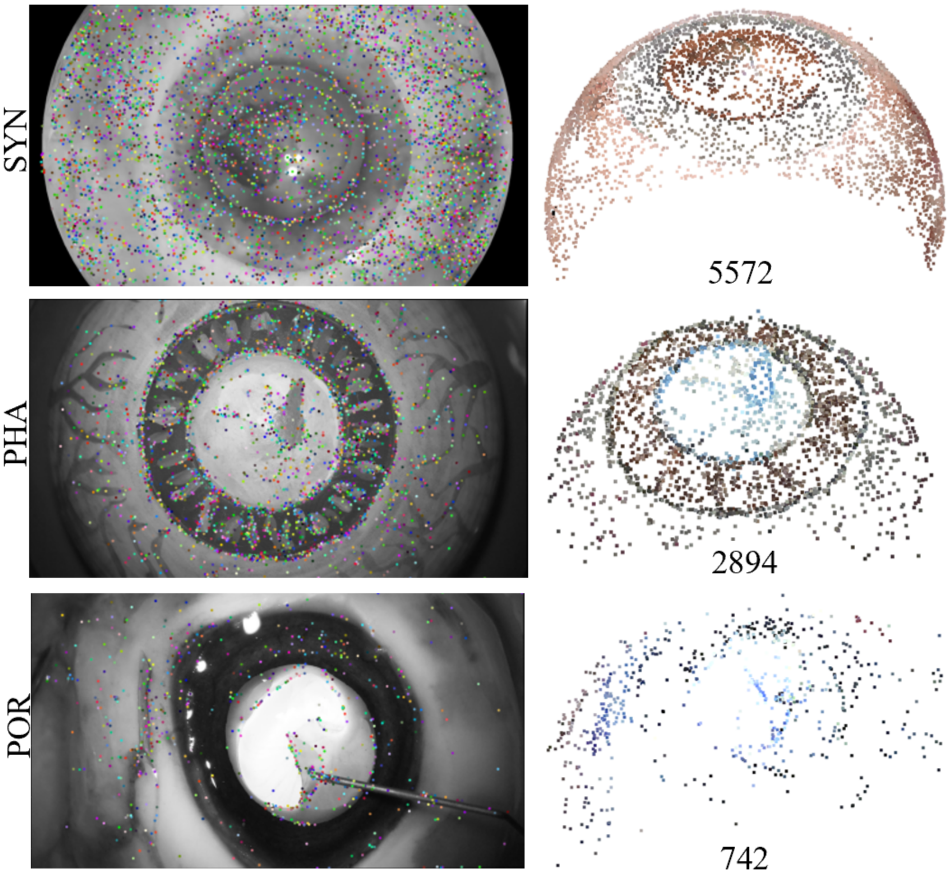

Online Resource 4: Identified correspondences in exemplary synthetic (SYN), phantom eye (PHA) and ex-vivo porcine eye (POR) images and their associated sparse point cloud with number of correspondences. Dependent on the level of texture and contrast, the number of correspondences is the highest for the photo-realistic SYN data (visually most similar to human eyes) and the lowest for POR (lowest contrast). The accuracy of stereo reconstruction is assumed to improve with the number of accurately matched correspondences.
